# Clinical Lessons From a Case of Retained Non-absorbable Suture After Uterine Compression

**DOI:** 10.7759/cureus.84575

**Published:** 2025-05-21

**Authors:** Ken Takahashi, Nozomu Yanaihara, Hiroshi Kishi, Osamu Samura, Aikou Okamoto

**Affiliations:** 1 Department of Obstetrics and Gynecology, The Jikei University School of Medicine, Tokyo, JPN

**Keywords:** complication, non-absorbable suture, non-absorbable thread, postpartum hemorrhage, uterine atony, uterine compression suture

## Abstract

Postpartum bleeding is commonly addressed with uterine compression using absorbable sutures; however, possible complications associated with non-absorbable threads are not well documented. We present a case in which a non-absorbable thread was mistakenly used to perform uterine compression sutures to treat postpartum uterine bleeding. Non-invasive methods, such as magnetic resonance imaging and hysteroscopy, failed to assess the sutures. As the patient wanted to have another child, diagnostic laparoscopy was performed one year later; the suture thread was still present, and a gap had formed between the thread and uterus, posing a risk for intestinal obstruction. The suture thread was successfully removed, and fertility treatment was resumed. This case highlights the dangers of using a non-absorbable thread for uterine compression sutures and the importance of promptly removing these sutures to avoid complications. Simulation training and pre-prepared suture materials are essential to prevent such errors. This case highlights the clinical risks associated with the use of non-absorbable sutures for uterine compression and the necessity of prompt identification and intervention to protect reproductive health.

## Introduction

Postpartum hemorrhage (PPH) remains a leading cause of maternal morbidity and mortality worldwide [1]. Among the various causes of PPH, uterine atony remains the most common, accounting for approximately 70-80% of cases [2]. When treatments such as uterotonics fail, surgical interventions, including uterine compression sutures, are employed to preserve the uterus and prevent hysterectomy [3]. Among these, uterine compression sutures are a particularly effective technique that can help achieve hemostasis by strongly compressing the anterior and posterior walls of the uterus, thereby preventing heavy bleeding due to uterine atony after childbirth [3,4]. However, complications such as Asherman’s syndrome [5] and pelvic adhesions [6] have been reported following uterine compression suturing, and unnecessary implementation should be avoided. Absorbable sutures are generally used for uterine compression suturing [3,4]. Furthermore, the use of specialized absorbable sutures has been recommended to prevent complications and achieve sufficient uterine compression suture effects [7]. However, few studies have investigated the adverse events associated with the use of non-absorbable threads when performing uterine compression suturing. Here, we present a case of a 35-year-old woman who mistakenly received uterine compression sutures using non-absorbable threads for the treatment of uterine atony.

## Case presentation

A 35-year-old woman with no significant medical history became pregnant for the first time via in vitro fertilization. She had been receiving antenatal care at Jikei University Hospital since the early stages of pregnancy. The pregnancy progressed well, and in the 40th week of gestation, a planned delivery using oxytocin was performed. Labor was induced at 40 weeks and 0 days with intravenous oxytocin due to prolonged gestation. However, despite adequate uterine contractions occurring every two to three minutes lasting over three hours, cervical dilation failed to progress beyond 5 cm. Therefore, delivery arrest was confirmed, and an emergency cesarean section was performed. A transverse incision was made in the lower part of the uterus, and the infant was delivered smoothly. The newborn weighed 2,725 g (appropriate for gestational age) and had an Apgar score of 8/9. The cord arterial blood gas pH was 7.353. The placenta was delivered smoothly. Subsequently, the uterus contracted poorly, and although oxytocin and methylergometrine were administered, sufficient uterine contractions were not achieved. At this point, the differential diagnoses included uterine atony and retained placental tissue. However, complete and intact delivery of the placenta, coupled with a normal uterine contour on inspection, ruled out retained placental tissue. Therefore, uterine atony was established as the primary etiology of the hemorrhage. Hence, balloon tamponade was performed; however, the balloon slipped and was ineffective. Given the challenges encountered during labor and delivery, further intervention was warranted.

**Figure 1:**
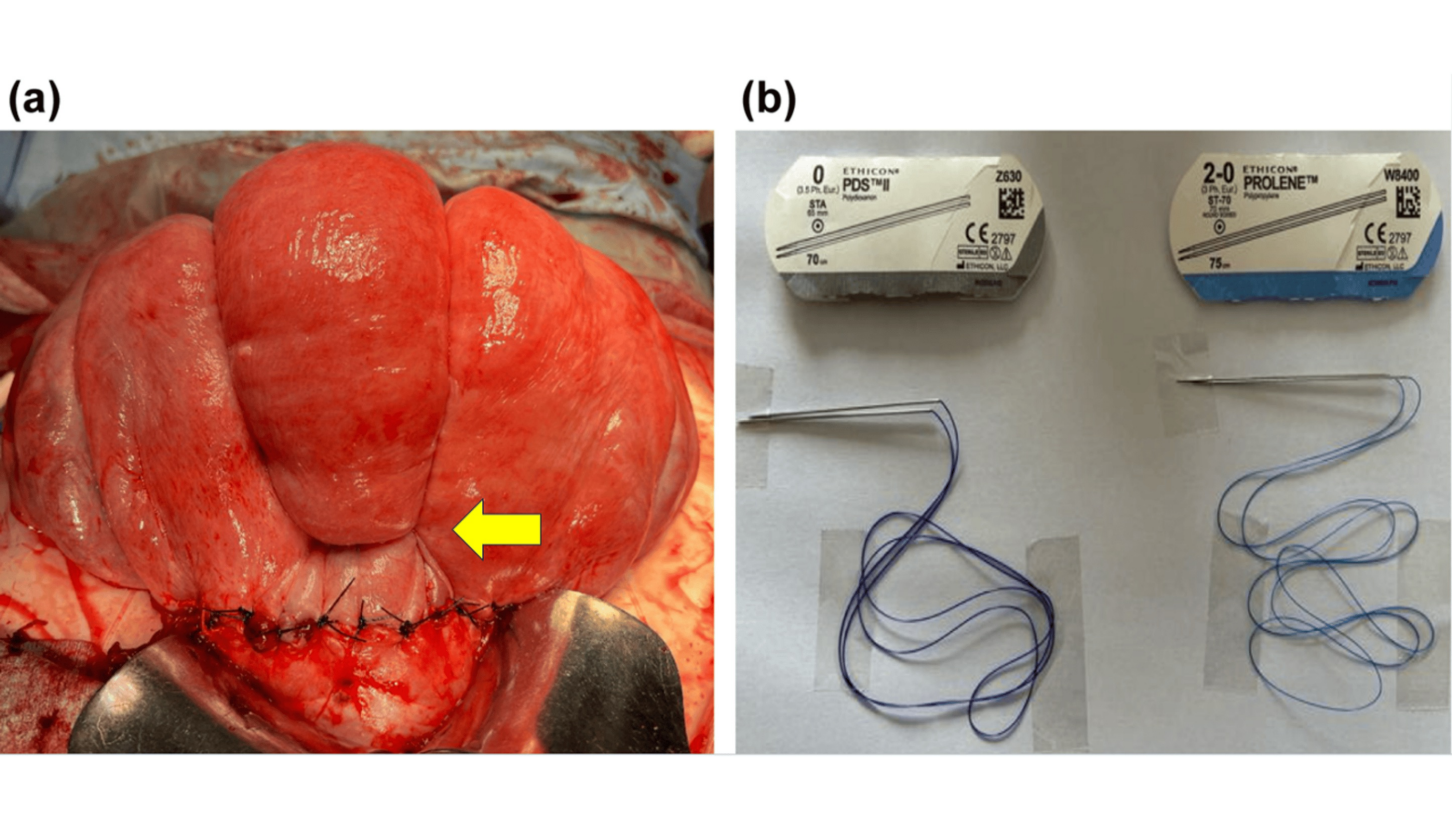
Uterine compression suture using a non-absorbable thread. (a) Uterine compression suture technique partially based on the Matsubara–Yano method. A needle was inserted through the anterior uterine wall slightly above the uterine incision and passed through the posterior wall, and a strong compression suture was placed at the uterine fundus to compress the uterus. The procedure was performed bilaterally. To prevent the sutures from slipping laterally, an additional suture was placed in the lower uterine segment (yellow arrow). No sutures were applied to the upper uterine body, as the myometrial layer in this area was too thick to allow safe needle passage. (b) Absorbable thread (left) and non-absorbable thread (right). Both had similar straight needles.

To address the observed complications, we implemented targeted diagnostic and therapeutic interventions. At this point, the amount of bleeding exceeded 3,000 ml. Bleeding increased rapidly post-placental expulsion and was refractory to uterotonics or balloon tamponade, necessitating surgical intervention. Therefore, uterine compression suturing was performed. The needle could not pass through the thick anterior and posterior walls of the uterine body; thus, uterine compression suturing was performed using an improved version of the Matsubara-Yano method. A horizontal suture was added to prevent the fundus of the uterus from shifting to the left or the right. However, a horizontal suture was not placed on the fundus side of the uterus because the myometrium was too thick for the suture to penetrate effectively (Figure 1A). The patient lost 3,135 ml of blood but did not develop disseminated intravascular coagulation due to sufficient blood transfusion.

On the day after surgery, after checking the suture thread used for the uterine compression procedure, we found that a non-absorbable thread (straight needle 2-0 Prolene, Ethicon, Raritan, NJ) was used instead of an absorbable thread (straight needle, 0 polydioxanone (PDS) Ethicon) (Figure 1B). Both threads have similar straight needles, and at our hospital, the supply stock contains both types of threads, which likely explains this error. Although the use of non-absorbable thread carries risks such as uterine infection, necrosis, intestinal obstruction, and other unexpected complications, we opted to monitor the patient rather than proceed with another surgery to reduce the patient’s surgical burden. We hoped that the non-absorbable sutures would loosen naturally and detach over time. We explained the situation to the patient and reported it to the hospital’s medical safety department. With close monitoring and patient safety in mind, the next step was to evaluate the patient’s progress postoperatively.

**Figure 2:**
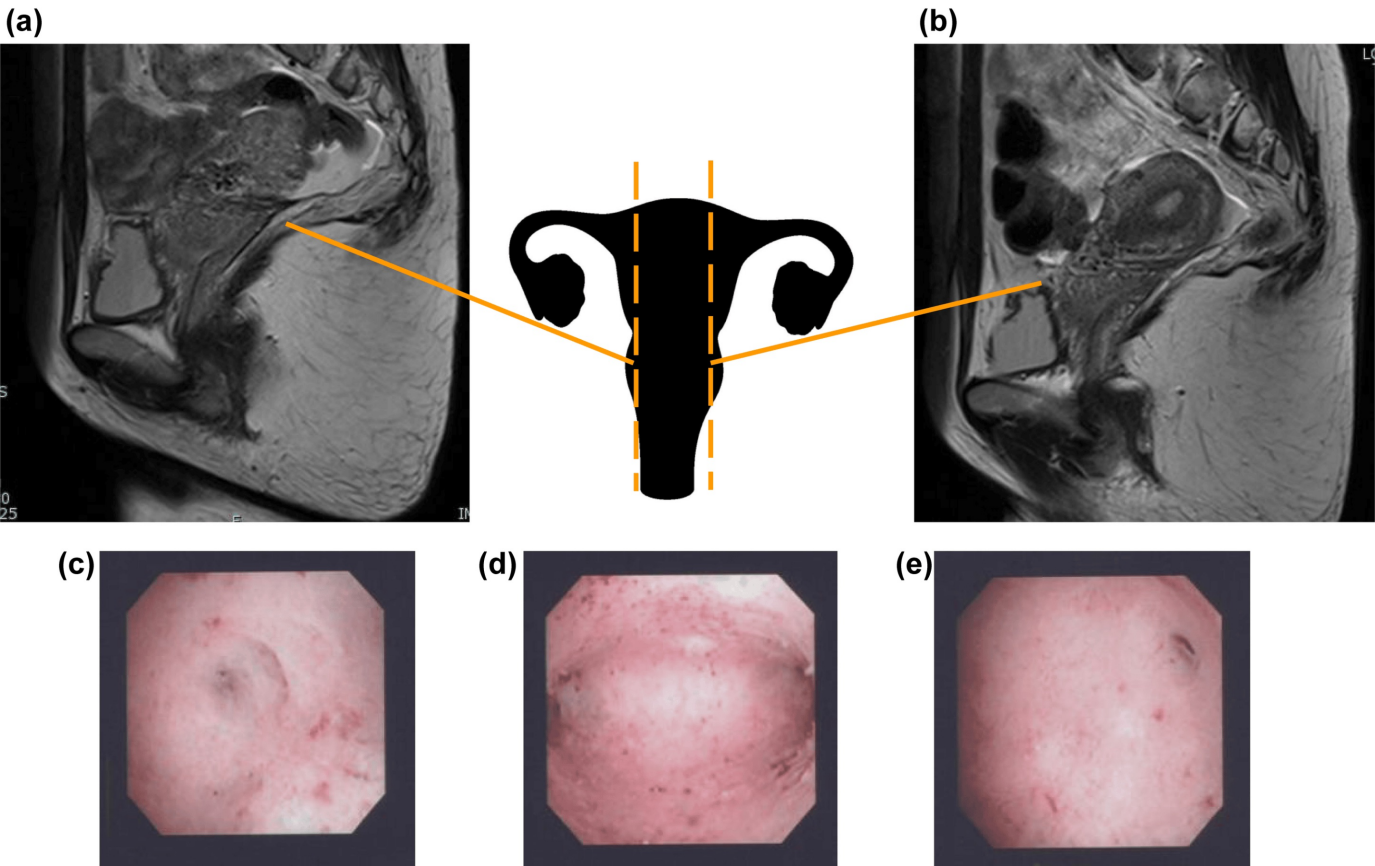
Evaluation of non-absorbable sutures using non-invasive methods. (a, b) T2-weighted MRI images six months postoperatively. Low-signal images indicating hemosiderin deposition were observed at the locations where the compression sutures had been placed; however, the sutures were not visible. (c-e) Hysteroscopic images taken one year postoperatively. (c) No obvious abnormal findings were observed in the area around the right fallopian tube opening. (d) No adhesions or sutures were observed in the uterine cavity. (e) No obvious abnormalities were observed in the area around the left fallopian tube opening.

After the postpartum period, no abnormalities such as abdominal pain, menstrual abnormalities, or signs of infection were observed. If the patient had no desire to have another child, we would have considered the option of continuing to monitor her condition; however, since she wanted to have a second child, a change in the management strategy was warranted. If non-absorbable threads are left in place, there is a risk of the expanding pregnant uterus tearing the myometrium, leading to severe bleeding during pregnancy. The condition of the thread must be evaluated to ensure that the patient can conceive. We preferred non-invasive evaluation of the suture thread; therefore, magnetic resonance imaging (MRI) was performed six months postoperatively, and low signals reflecting hemosiderin deposition, corresponding to the position of the uterine compression suture thread, were confirmed (Figures 2A, 2B). However, these signals were non-specific and could be interpreted as postoperative changes, making it difficult to determine whether the thread was still present.

**Figure 3:**
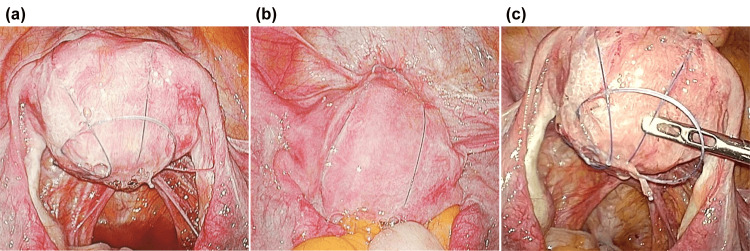
Findings of diagnostic laparoscopy. (a, b) The thread remained in the same condition as it was at the time of application. (c) A large space was formed between the uterus and the suture thread, posing a risk of the intestinal tract becoming embedded within it.

To allow the uterus sufficient time to recover, the patient was advised to avoid pregnancy for at least one year after the cesarean section. However, after one year, it became necessary to evaluate whether the patient’s condition was suitable for pregnancy. Because uterine compression suturing was performed such that the sutures penetrated the uterine cavity, we thought that the sutures could be detected via hysteroscopy. However, hysteroscopy performed one year postoperatively showed no obvious sutures or adhesions in the uterine cavity (Figures 2C-2E). After extensive discussion, it was determined that prioritizing patient safety was essential. Consequently, after thoroughly explaining the conclusions to the patient, diagnostic laparoscopy was performed two months later. We found that the sutures had not yet come undone and that there was a large space between the sutures and the uterus due to the uterus shrinking back to its pre-pregnancy size. This space was considered dangerous because it posed a risk of intestinal tract obstruction (Figure 3). The sutures were removed without any complications during laparoscopy. Since the patient’s postoperative course was favorable, she was considered suitable for subsequent pregnancy. Although she is currently undergoing infertility treatment, there were no obvious intra-abdominal adhesions on laparoscopy, and Asherman syndrome was not observed on hysteroscopy. Therefore, the non-absorbable thread used for uterine compression sutures is unlikely to have affected the results of infertility treatment.

This study was approved by the Ethics Committee of Jikei Hospital (IRB number: 36-069(12168)) and adhered to the principles of the Declaration of Helsinki. Written informed consent was obtained from the patient for publication of this case report.

## Discussion

Herein, we describe a case in which we mistakenly used non-absorbable sutures rather than the recommended absorbable sutures for uterine compression in a life-threatening case of postpartum hemorrhage. In this case, we learned two important points. First, when non-absorbable sutures are used for uterine compression, it is difficult to determine whether the sutures have been retained using non-invasive methods. Second, even after a year, the sutures may fail to come undone and cause serious complications, such as intestinal obstruction. Therefore, when a non-absorbable thread is used for uterine compression suturing, the suture must be removed promptly.

Initially, we expected the non-absorbable thread to unravel and naturally fall into the abdominal cavity or uterus. However, because the patient wished for another pregnancy, the condition of the non-absorbable thread needed to be evaluated. Metallic sutures can cause artifacts during MRI [8], and threads with special coating technology can be visualized using MRI [9]. However, because the non-absorbable thread used in the present case was non-metallic, it could not be detected. Only non-specific findings suggestive of hemosiderin deposition were observed. In addition, hysteroscopy is commonly performed to evaluate the uterine cavity and is well known for its high accuracy [10]; however, the presence or absence of suture thread could not be confirmed in the present case. We assumed that the thread had already come undone and had fallen into the abdominal cavity because the part of the uterus penetrated by the uterine compression suture was not in the uterine cavity and could not be accurately assessed using hysteroscopy. Therefore, non-invasive tests cannot accurately evaluate the non-absorbable sutures used for uterine compression.

A laparoscopic examination was performed one year and two months after surgery to check the status of the non-absorbable thread. The thread remained in the same condition as when it was initially applied. Furthermore, we observed a dangerous gap between the thread and the uterus, in which the intestinal tract could potentially become trapped. Although no clear adhesions were observed in the abdominal cavity, the risk of adhesions was considered high [11]. If uterine compression is mistakenly performed using non-absorbable sutures, immediate removal should be considered. A few previous reports have highlighted the risks associated with the use of non-absorbable sutures for uterine compression, including persistent pelvic pain and the need for reoperation [12,13]. These findings support our concern that retained non-absorbable sutures may lead to serious postoperative complications if not promptly addressed. To prevent such mistakes in the future, simulation training that envisages the situation in which uterine compression suturing is performed must be provided, and the necessary equipment should be prepared in advance. No formal scoring system or clinical assessment scale was used in this case. Clinical decisions were made based on the patient’s symptoms, physical findings, imaging results, and overall clinical course.

## Conclusions

In conclusion, we describe the case of a woman who mistakenly underwent uterine compression suturing using a non-absorbable thread. More than a year after surgery, not only did the non-absorbable sutures not dissolve or loosen, but a large space was also formed between the sutures and uterus, which could potentially result in the intestinal tract becoming trapped within the space. In addition, the sutures could not be accurately assessed using non-invasive methods. The lessons learned from the present case are useful for obstetricians who may encounter similar situations and for establishing measures to prevent similar mistakes.

## References

[1] Say L, Chou D, Gemmill A (2014). Global causes of maternal death: a WHO systematic analysis. Lancet Glob Health.

[2] Evensen A, Anderson JM, Fontaine P (2017). Postpartum hemorrhage: prevention and treatment. Am Fam Physician.

[3] B-Lynch C, Coker A, Lawal AH, Abu J, Cowen MJ (1997). The B-Lynch surgical technique for the control of massive postpartum haemorrhage: an alternative to hysterectomy? Five cases reported. Br J Obstet Gynaecol.

[4] Matsubara S, Yano H, Taneichi A, Suzuki M (2009). Uterine compression suture against impending recurrence of uterine inversion immediately after laparotomy repositioning. J Obstet Gynaecol Res.

[5] Kwong LT, Wong SF, So PL (2023). Menstrual, fertility and psychological impacts after uterine compression sutures for postpartum hemorrhage: a prospective cohort study. BMC Pregnancy Childbirth.

[6] Ramler PI, Henriquez DDCA, van den Akker T (2020). Comparison of outcome between intrauterine balloon tamponade and uterine artery embolization in the management of persistent postpartum hemorrhage: a propensity score-matched cohort study. Obstetric Anesthesia Digest.

[7] Matsuzaki S, Endo M, Tomimatsu T (2019). New dedicated blunt straight needles and sutures for uterine compression sutures: a retrospective study and literature review. BMC Surg.

[8] Leitgeb N, Loos G, Ebner F (2013). MRI-induced tissue heating at metallic sutures (cerclages). J Electromagn Anal Appl.

[9] Thierry B, Faghihi S, Torab L, Pike GB, Tabrizian M (2005). Magnetic resonance signal‐enhancing self‐assembled coating for endovascular devices. Adv Mater.

[10] Curlin H, Cholkeri-Singh A, Leal JG, Anderson T (2022). Hysteroscopic access and uterine cavity evaluation 12 months after endometrial ablation with the cerene cryotherapy device. J Minim Invasive Gynecol.

[11] An GH, Ryu HM, Kim MY, Han JY, Chung JH, Kim MH (2013). Outcomes of subsequent pregnancies after uterine compression sutures for postpartum hemorrhage. Obstet Gynecol.

[12] Cotzias C, Girling J (2005). Uterine compression suture without hysterotomy--why a non-absorbable suture should be avoided. J Obstet Gynaecol.

[13] Cengiz SD, Çağlar GS, Gürsoy AY, Kiseli M, Yılmaz B (2016). Adverse events after uterine compression sutures for postpartum hemorrhage: report of three cases and review of the literature. Gynecol Obstet Reprod Med.

